# Supplementary material for: Nitrogen Supply Drives Senescence-Related Seed Storage Protein Expression in Rapeseed Leaves
Source: Genes (Basel). 2019 Jan 22;10(2):72. doi: 10.3390/genes10020072 (PMC6410074; doi:10.3390/genes10020072)
Supplement: Supplementary file 1 [file genes-10-00072-s001.zip › Supporting infomation-Bieker et al.-Genes.pdf]

Seed Storage Protein Expression in Vegetative Tissue is driven by Nitrogen Supply during Senescence in Oil Seed Rape Plants

Stefan Bieker[1], Lena Riester[1], Jasmin Doll[1], Jürgen Franzaring[2], Andreas Fangmeier[2], Ulrike Zentgraf[1*]

[1]Centre of Molecular Biology of Plants, University of Tübingen, Auf der Morgenstelle 32, D-72076 Tübingen, Germany
[2] Institute of Landscape and Plant Ecology, University of Hohenheim, August-von-Hartmann-Str. 3, D-70599 Stuttgart, Germany

Correspondence:
*Ulrike Zentgraf
ulrike.zentgraf@zmbp.uni-tuebingen.de

Supporting Figures

Supplementary Figure S1

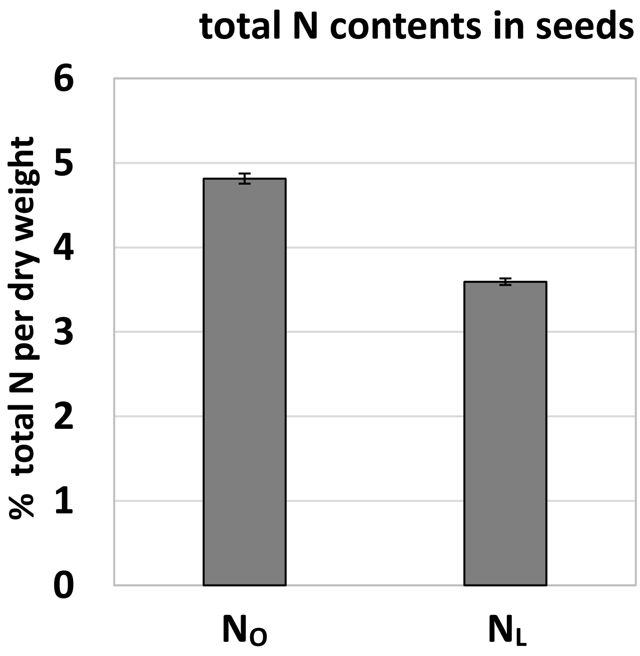

Figure S1. *Total N content in seeds.*
Total N content was measured by a CN-element analyser in % of the dry weight of the seeds collected from plants grown under N_L and N_O conditions. Error bars represent *SD* of two biological replicates.

Supplementary Figure S2

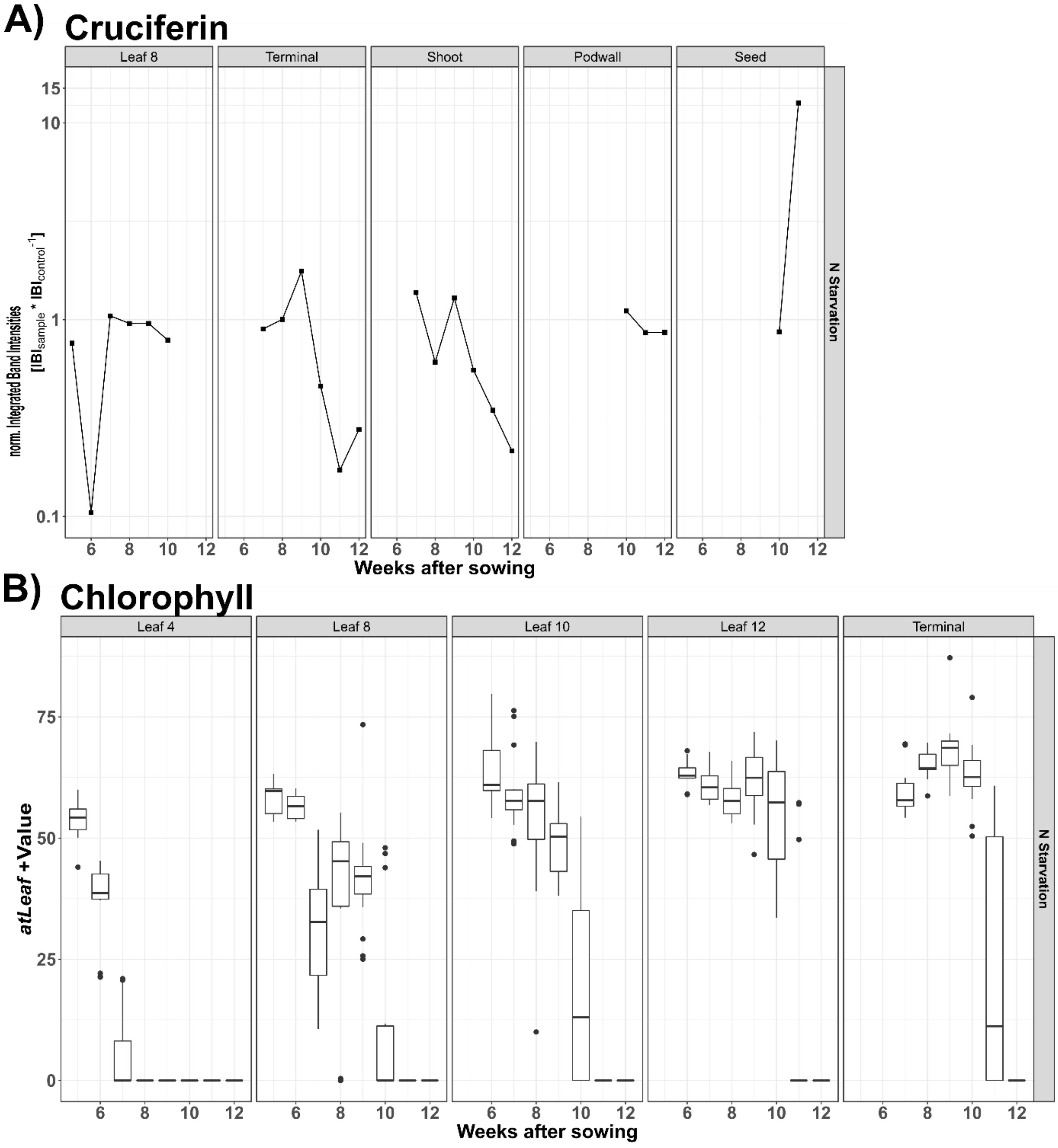

Figure S2. *Prolonged N-Starvation in B. napus.*
A) Quantification of immune-detection of CRU proteins in material from OSR (cv. Mozart) plants grown under N-starvation conditions. Accumulation of protein is barely detectable in leaf No. 8, the terminal leaf and the shoot show minimal build-ups. Seed extracts display highest accumulation of CRU proteins. **B)** Corresponding chlorophyll contents of different leaf positions of N-starved plants. Between week 5 and 7 values decreased at all analysed leaf positions. In the following weeks, leaf No. 4 is shed, all other positions exhibit an interruption of the decline or even an increase in chlorophyll content followed by a final rapid decrease before shedding. Chlorophyll contents are shown as median +/- 1.5xIQR of 3 biological replicates.

Supplementary Figure S3

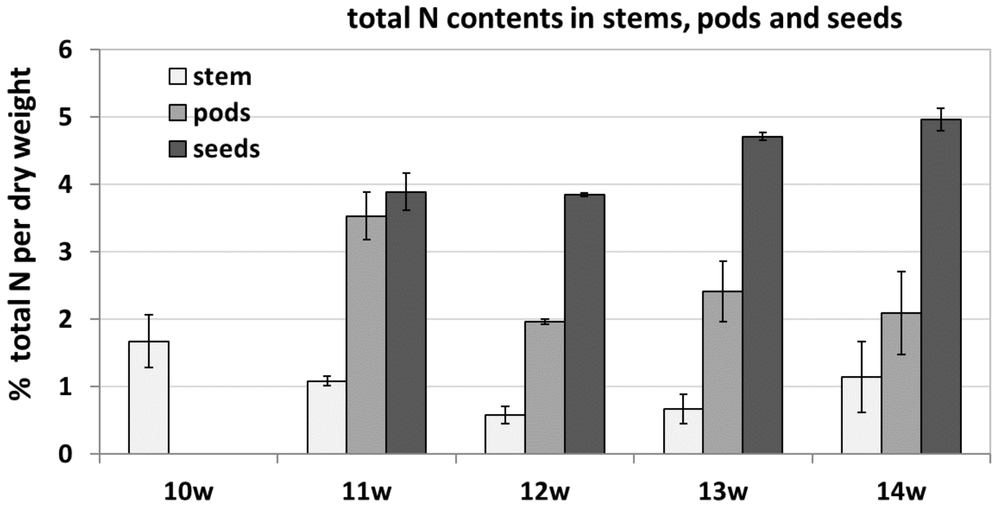

Figure S3. *Total N contents of stems pods and seeds.*
Total N content was measured by a CN-element analyzer in % of the dry weight of the respective tissue, stem, pods and seeds over development from week 10 to week 14. Error bars represent *SD* of two biological replicates.

Supplementary Figure S4

Cruciferin

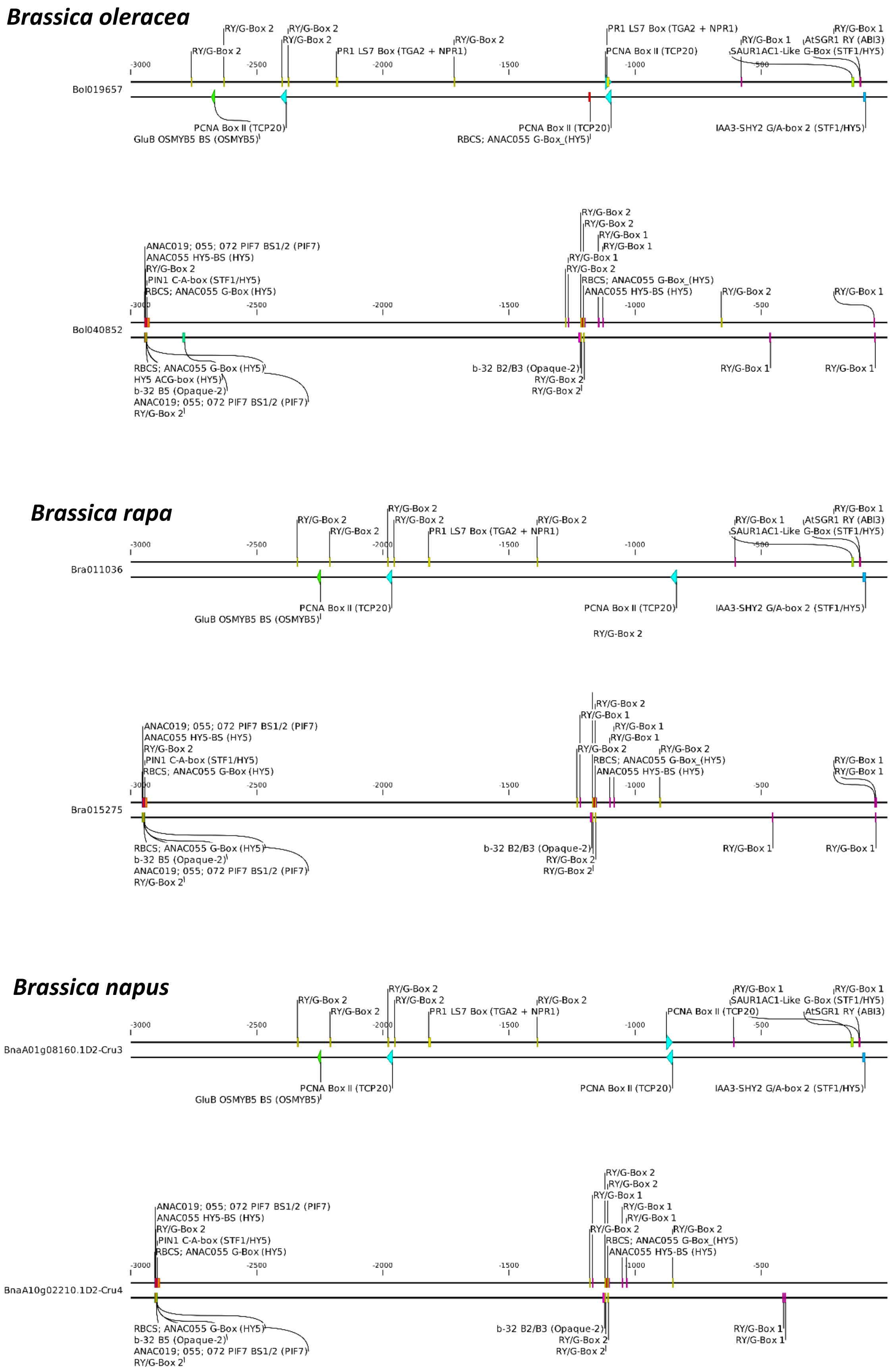

Napin

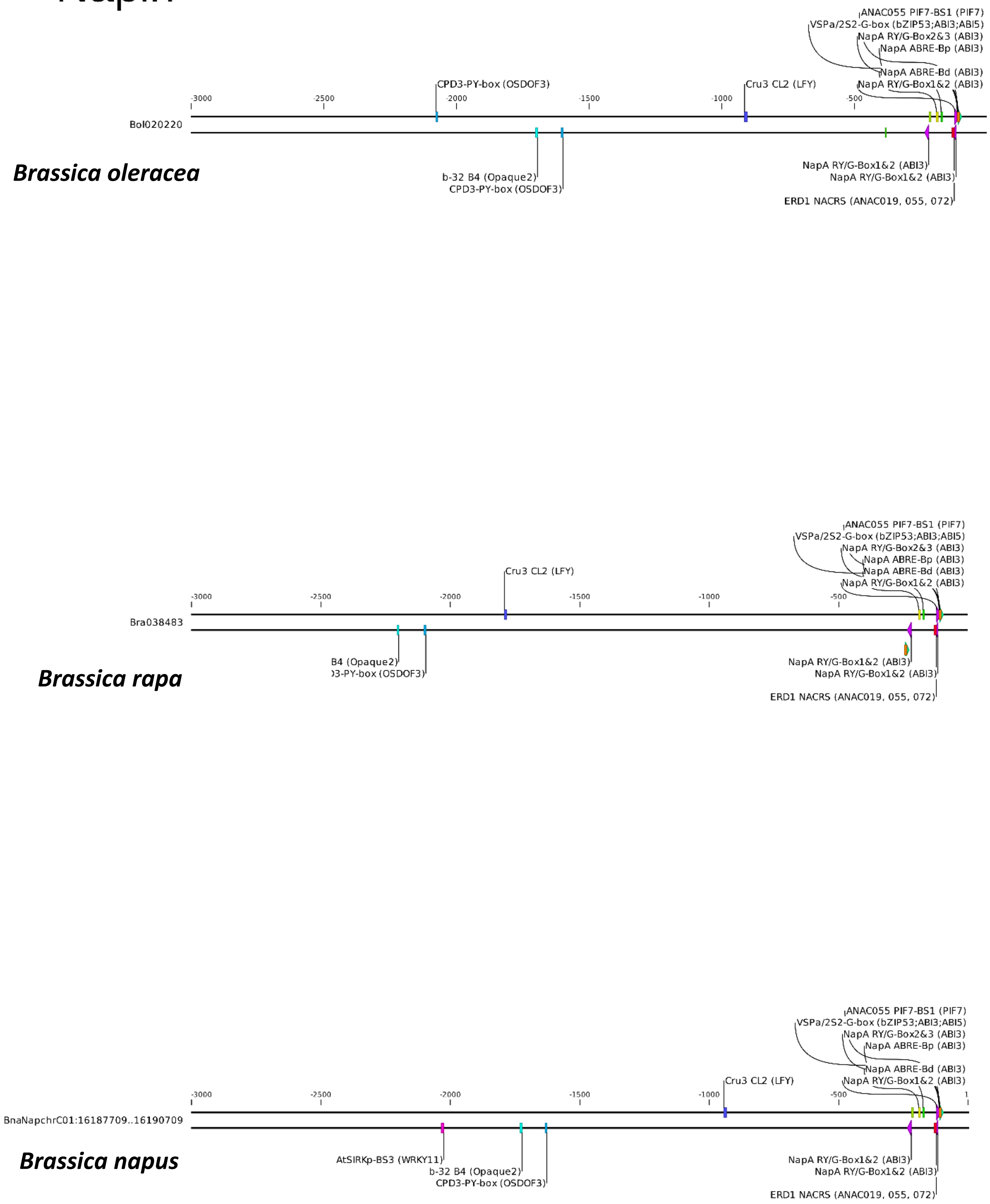

Figure S4. *Seed storage protein (SSP) promotor structure and identified binding elements***.** Oilseed rape (Brassica napus L.) was formed ~7500 years ago by hybridization between *B. rapa* and *B. oleracea*, followed by chromosome doubling. Therefore, 3kb upstream of *B. rapa* (top) and *B. oleracea* (middle) as well as *B. napus* (bottom) *NAPIN* and *CRUCIFERIN* genomic sequences were analysed for *cis* elements. Depictions generated with CLC Main Workbench.

Supplementary Figure S5

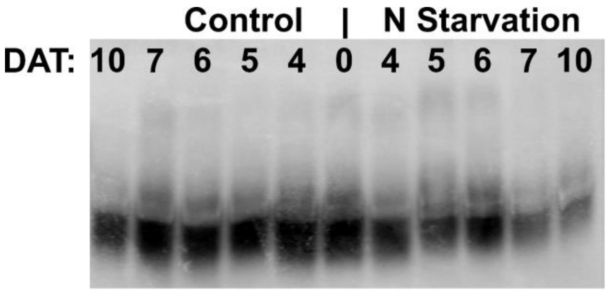

Figure S5: *Catalase (CAT) zymogram of nitrogen (N) starved OSR plants (cv. NPZ1).* CAT activity declines in N-starved plants from 5 DAT on (right), whereas control plants exhibit only minor variations in CAT activity over time (left). Media for N-starvation see Table S2. DAT: days after treatment. Image of CAT zymogram was transformed to grayscale and inverted.
